# FSRW: fuzzy logic-based whale optimization algorithm for trust-aware routing in IoT-based healthcare

**DOI:** 10.1038/s41598-024-66392-4

**Published:** 2024-07-18

**Authors:** Hui Xu, Wei-dong Liu, Lu Li, Deng-ju Yao, Lin Ma

**Affiliations:** 1 Heilongjiang University of Chinese Medicine, Harbin, 150040 China; 2 Heilongjiang Provincial Big Data Center of Government Affairs, Harbin, 150028 China; 3 Harbin University of Science and Technology, Harbin, 150006 China

**Keywords:** Internet of Things, Healthcare, Routing, Fuzzy logic, Whale optimization algorithm, Engineering, Mathematics and computing

## Abstract

The Internet of Things (IoT) is an extensive system of interrelated devices equipped with sensors to monitor and track real world objects, spanning several verticals, covering many different industries. The IoT's promise is capturing interest as its value in healthcare continues to grow, as it can overlay on top of challenges dealing with the rising burden of chronic disease management and an aging population. To address difficulties associated with IoT-enabled healthcare, we propose a secure routing protocol that combines a fuzzy logic system and the Whale Optimization Algorithm (WOA) hierarchically. The suggested method consists of two primary approaches: the fuzzy trust strategy and the WOA-inspired clustering methodology. The first methodology plays a critical role in determining the trustworthiness of connected IoT equipment. Furthermore, a WOA-based clustering framework is implemented. A fitness function assesses the likelihood of IoT devices acting as cluster heads. This formula considers factors such as centrality, range of communication, hop count, remaining energy, and trustworthiness. Compared with other algorithms, the proposed method outperformed them in terms of network lifespan, energy usage, and packet delivery ratio by 47%, 58%, and 17.7%, respectively.

## Introduction

The healthcare industry has experienced notable progress, resulting in large job prospects and increased revenue generation. Historically, the identification of diseases and abnormalities in the human body necessitated physical evaluations conducted in medical facilities, sometimes resulting in patients being admitted for treatment^1^. This not only increased healthcare expenses but also put pressure on rural and distant healthcare facilities. However, technological progress has transformed this landscape^2^. Numerous ailments can be diagnosed today, and health can be monitored using small devices like smartwatches. Using advanced technology, patients are now able to access and monitor their clinical parameters in their homes without the need for the support of clinicians. Telecommunications technology enables the secure transmission of data collected in remote regions to healthcare institutes^3^. Medical services and facilities have become significantly more accessible. The swift progress of technology has ushered in innovations, including big data analytics, machine learning, cloud computing, Internet of Things (IoT), and remote sensing^4,5^.

Modern healthcare has greatly benefited from worldwide technological advancements and biological developments in recent years^6^. Sensors are now ubiquitous in internet-connected devices, including automobiles^7^. Healthcare professionals in the healthcare industry are now integrating Internet of Medical Things (IoMT) technology to facilitate patient care and monitoring with the use of telemonitoring. IoT can process and make autonomous decisions on patient-generated data signals in real-time. A challenge is the privacy and security of personal data and potentially modified data during transit^8^.

Consequently, IoT devices require security measures that are lightweight, scalable, and capable of ensuring distributed privacy^9^. Decentralization means shifting control and authority from a centralized entity to a distributed system, which is frequently achieved through the adoption of blockchain technology^10^. Decentralized networks allow for an element of trust among users to a certain extent, which reduces the need for excessive authority or control that could disrupt the integrity of the system's overall performance^11^. Decentralized systems provide trust, data reconciliation, fewer vulnerabilities, optimal resource allocation, encryption efficiency, and secure storage and privacy of confidential or sensitive information^12–14^. Encryption is a response to the need for secure data storage and privacy; it serves to block unauthorized access to data and stop illegitimate usage of confidential data^15–17^.

Energy efficiency is a crucial aspect of IoT healthcare. Insufficient energy in sensor nodes can lead to reduced disease diagnosis accuracy and service interruptions^18^. Energy depletion is an issue that occurs when data passes through IoT objects and cloud servers and particularly affects nodes driven by batteries, which connect sensors, microcontrollers, and critical nodes in wearable IoT tools^19^. To tackle this issue, fog-based strategies and other methodologies are utilized to enhance energy efficiency to a considerable extent, all the while preserving the interconnectedness among the intelligent gateways and sensor nodes^20^. Within the architecture of IoT healthcare, the integration of IoT devices occurs through the formation of clusters, which serve the purpose of monitoring health information^21^. The selection of the proper channel for data transfer to the cloud server of IoT devices is facilitated through fog nodes^22^. IoT technology enables the digitized management of health records, affording medical personnel with increased opportunities to allocate their time toward patient care, ultimately resulting in enhanced medical services^23^.

Security assurance is the fundamental requirement in the IoT, from simple devices like sensors, given the plethora of devices present in the network^24,25^. Creating more robust and energy-efficient routing algorithms for secure data transfer in IoT health devices is challenging^26^. To tackle this issue, the present study introduces FSRW, a fuzzy secure hierarchical routing mechanism that utilizes the Whale Optimization Algorithm (WOA) within the context of Wireless Sensor Network (WSN)-based IoT networks. The main goal of the FSRW is to augment the network's security level while simultaneously enhancing energy efficiency. The relationship between security and energy consumption is generally characterized by an inverse correlation, meaning that when adequate security measures are implemented, there is usually an increase in energy consumption. Therefore, it is crucial to attain a balanced state in order to build a routing approach that is both secure and energy-efficient. The primary contributions of the FSRW are as follows:

- The FSRW approach presents a trust evaluation system rooted in fuzzy theory. Its purpose is to assess the reliability of IoT nodes and address cybersecurity threats that specifically target IoT networks. The present architecture has been specifically developed to identify and mitigate diverse forms of attacks, including but not limited to black holes, flooding, wormholes, sinkholes, and grey holes. To construct the trust framework, four scales are considered: consumed energy ratio, packet reception frequency, packet transfer frequency, and packet delivery ratio.
- The FSRW suggests a clustering method that employs the WOA to reduce energy usage in IoT nodes, decrease communication overhead, and alleviate network congestion. The approach to clustering integrates a novel objective function that considers the trust degree of IoT nodes to augment security in the network during the clustering procedure. Furthermore, the objective function takes into consideration energy levels and the hop counts between Cluster Heads (CHs) and the Base Station (BS), resulting in enhanced energy efficiency within the proposed approach.
- The FSRW strategy provides a novel routing method that seeks trustworthy and energy-efficient pathways among nodes and is designed to prevent incorrect pathways and risky routes within the network. This method involves only trustworthy nodes in the route location phase. In contrast, any nodes exhibiting hostile behavior are deliberately excluded from participating in the cooperative efforts.

## Related work

This section examines current research on healthcare systems utilizing the IoT, which includes several innovative methods to overcome numerous challenges in the industry. These challenges encompass the requirement for real-time health monitoring, the centralization of data, the enhancement of resource use, as well as accurate diagnostics and predictive analytics. As shown in Table 1, each work employs unique methodologies and applications aimed at advancing the provision of intelligent and effective healthcare services in the IoT era.

**Table 1:** Recent IoT-based Healthcare techniques.

Paper	Key contributions	Applications	Advantages	Shortcomings
Satpathy et al.^27^	IoT-based health analysis system with fuzzy classifier	Consumer electronic health monitoring	Higher accuracy in pathological condition indication	Limited scalability due to dependency on FPGA
		Real-time health monitoring	Reduced execution time	Potential privacy issues with cloud data storage
		Cloud-based data analysis		
Sajedi et al.^28^	F-LEACH data aggregation for IoT healthcare applications	Energy conservation	5%-20% increased network lifetime	Fuzzy logic may not adapt to dynamic changes effectively
Singh et al.^29^	New mutation operator integrated with DE algorithm	IoT-based smart healthcare systems	Faster search speed for optimization problems	High computational complexity
		IoT sensor deployment optimization	Minimization of function evaluations, traffic services, and energy loss	It may be reliant on frequent parameter tuning
Tyagi et al.^30^	Radial basis function of neural network for patient localization	Remote patient monitoring	Efficient and accurate patient localization outside of cellular coverage	Dependent on ongoing sensor connectivity
		Real-time prediction		
Arivazhagan et al.^31^	Smart model for task scheduling in HMFO framework	Cloud-based healthcare applications	Improved resource assignment, enhanced QoS, and reduced response time	Complexity in real-time deployment
		Hybrid cloud environment	Minimization of overall costs and run time	Potential overhead with hybrid framework management
Kanna et al.^32^	Firefly algorithm for optimizing maize crop yield	Agricultural optimization	Precise prediction of optimal climatic conditions	Dependent on long-term climatic data
		Cloud-based data storage	Accurate forecast of crop output	
Irshad et al.^33^	IoT-enabled lung cancer detection platform	Lung cancer diagnosis	Enhanced accuracy in lung cancer diagnosis	Strong dependency on training data quality
		IoT-based healthcare platform	Superiority over state-of-the-art lung cancer detection models	Potential for overfitting in neural network models

Satpathy et al.^27^ have proposed an innovative IoT-based analysis system for designing a consumer electronic device. This system monitors the user's health parameters and promptly alerts them if any of the parameters fall outside the acceptable threshold. The records acquired are transmitted to the cloud via mobile applications and analyzed by a field-programmable gate array (FPGA) algorithm. The processed data would be displayed on patients' wearable devices to provide information about potential pathological issues. The primary advancement of this study lies in the utilization of a fuzzy classifier, which outperforms naive Bayes, SVM, kNN, and decision trees in accurately identifying pathological conditions. Additionally, the execution time is reduced by implementing the fuzzy classifier on FPGA, enabling real-time health monitoring.

A novel data aggregation algorithm named F-LEACH was proposed by Sajedi et al.^28^ using fuzzy logic. It aims to maximize the lifetime of the network to enhance all IoT-based healthcare applications. The data aggregation process is crucial for IoT networks in order to combine the data from multiple sensors to decrease redundancy and save energy. The scheme was evaluated by the researchers through extensive simulations and compared to the performance of similar existing schemes. The researchers determined that F-LEACH's network lifespan performance was superior to the examined schemes by a margin of at least 5%-20%.

Singh et al.^29^ have developed a novel mutation operator incorporating the differential evolution (DE) algorithm. The novel dual adaption-based operators, when paired with DE, have the objective of improving the efficiency of searching for solutions in both local and global search scenarios. This approach has been proposed and validated with IoT-based smart healthcare systems applications. The first validation test confirms the suggested approach's value in delivering adequate variety and boosting search duration for optimization tasks. The method is especially useful for IoT sensor deployment in smart healthcare systems to minimize operation duration, delay, and wasted energy while considering communication restrictions between sensors (objects). The results reveal that the suggested approach outperforms typical evolutionary algorithms in IoT applications by requiring fewer functional executions and using fewer traffic resources. Furthermore, the strategy effectively limits energy usage in each service, lowering load and system delay.

Tyagi et al.^30^ have introduced a novel mechanism to detect patients' positions based on the radial basis function of the neural network. They use the radial basis function neural network to create the new approach. The purpose is to monitor the patient's health and provide instant health assessment. The main aim is to revolutionize healthcare and create an intelligent health system to offer the highest service quality. One possible application that could benefit from the proposed approach is remote patient monitoring. In this scenario, IoT sensors using batteries will collect the patient's health data, and then such data will be uploaded to the cloud for the doctor to watch the patient's health status. The researchers have synthesized the proposed technique by conducting many simulation studies and numeric analyses of realistic biomedical network setups to evaluate the method's applicability. The results from the evaluation show that the patient localization prediction is accurate and efficient which suggests the potential for the new approach in the intelligent systems suggested by the authors.

Arivazhagan et al.^31^ have proposed a novel method for optimizing task scheduling in a Hybrid Moth Flame Optimization (HMFO) framework for cloud computing that integrates the Internet of Health Things (IoHT) concept into electronic health records. The objective of this approach is to arrange the resources uniformly in order to boost the quality of service (QoS) for healthcare applications based on the cloud. The authors employed data from the Google cluster dataset in order to train the model, allowing the model to understand how jobs are assigned and scheduled in the cloud. Once the model is trained, real-time job scheduling can use HMFO to efficiently schedule jobs, taking into account different requirements and parameters. CloudSim was used for the simulation, where we evaluated the effectiveness of the hybrid HMFO approach for scheduling in the context of a hybrid cloud environment. The performance assessment of the model considered several important and predominant factors in the metrics, for example, resource utilization, energy consumption, and response time. Evaluation results indicate that the hybrid HMFO method benefits from runtime, cost, and response time compared to other techniques. The hybrid HMFO approach can also optimize task scheduling in the cloud-based health system. While achieving the same response rate, cost, and runtime, it was also able to decrease the overall cost and runtime.

Kanna et al.^32^ observed the effectiveness of the firefly algorithm in enhancing maize crop productivity under various limitations and hazardous conditions. Their study aimed to implement a firefly algorithm module to estimate crop yield and to anticipate the possible favorable environmental conditions for the maize plants. The researchers employed 96 months of data on maize crop yields. The receipts were associated and investigated by the Minitab software, and the relational equation was exploited. Then, the data from the corn crops were stored in the cloud, and an IoT application updated the cloud to consistently insinuate fair outcomes that could be acquired through the firefly algorithm. The relational equation from the data was the fitness function in the firefly algorithm. The firefly algorithm identified the possible most favorable conditions, such as the volumes of rainfall, availability of irrigation resources, and air temperature, by considering the key variables and the relation between the variables that lead to the highest maize crop production. The module they designed could predict the yield of maize crops precisely and have promising prospects for the predictability of different types of crops in the future.

Irshad et al.^33^ built a distinct IoT-enabled health monitoring system for lung cancer detection. A Deep Convolution Neural Network (DCNN) model and an improved Grey Wolf Optimization (GWO) algorithm have been integrated to improve lung cancer diagnoses. Specifically, lung nodules are diagnosed utilizing the Tasmanian Devil Optimization (TDO) algorithm. The original GWO algorithm's convergence rate is enhanced to create an improved version that enhances the optimization process. The IGWO-based DCNN model follows training on the selected feature set extracted from the IoT platform. The trained model has been developed to detect and diagnose lung cancer with 97.3% accuracy. The results are stored in the cloud for viewing and analyzing by experts. The system runs on an Android platform and working Python libraries with a Deep Convolutional Neural Network capable of detecting objects in images. The performance of the proposed method was compared with previous studies. This comparison demonstrated that the proposed method improves lung cancer detection compared with current methods.

The present study suggests a novel framework, FSRW, that combines fuzzy trust assessment and WOA for efficient and secure routing in IoT-based healthcare systems. Unlike previous works such as^27^, which focus on health monitoring and early detection using classifiers, FSRW specifically addresses routing security and energy optimization. Furthermore, while Sajedi et al. propose F-LEACH to improve network lifespan through data aggregation, FSRW incorporates trust assessment and CH selection for a holistic approach. Moreover, our approach differs from other methods by uniquely addressing routing security and energy efficiency in IoT healthcare networks.

## Backgrounds

This section examines FSRW's methodologies, including the WOA, a nature-inspired optimization algorithm, and fuzzy logic. Table 2 lists the existing symbols and their definitions used throughout the study.

**Table 2:** Main symbols used and descriptions.

Symbol	Definition	Symbol	Definition
*X*	Position vector	*a*	The coefficient vector gradually diminishes from 2 to 0
*C*	Coefficient vector	*r*	Unpredictable vector changing from 0 to 1
*A*	Coefficient vector	*p*	A variable representing randomness, ranging from 0 to 1
*t*	Current iteration	*S*	Solution found near a potential optimal solution
X*	Position vector of the best solution	*U*	Total number of nodes in the network
*BS*	Base station	*CH*	Cluster head
*CM*	Cluster member	*m*_*i*_	i^th^ node
*E*_*tx*_	Energy consumption for data transmission	*l*	Data size being exchanged
*E*_*rx*_	Energy consumption for data reception	*E*_*elec*_	Energy expended by the transceiver circuitry
*ε*_*fs*_	Energy consumed for free space model	*ε*_*mp*_	Energy consumed for multi-path fading model
*d*	Distance between the transmitter and receiver nodes	*d*_*0*_	Threshold distance
${T}_{ij}^{direct}$	Urgent trust generated by the *i*^*th*^ node for the *j*^*th*^ node	*ECR*_*j*_	The energy consumption ratio of node j
*E*_*j*_*(t)*	Remaining energy of node j at time t	*PRF*_*j*_	Number of packets received by node j
${M}_{j}^{Received}$	Count of packets received by the j^th^ node	*PTF*_*j*_	Packet count sent by node j
${M}_{j}^{Transferred}$	Total packet count sent by node j	*D*_*avg*_	Average distance to nearby nodes
${\beta }_{1},{\beta }_{2},{\beta }_{3}, and {\beta }_{4}$	Weight coefficients adjusted within the range of [0, 1]	${\omega }_{1}, {\omega }_{2},{\omega }_{3},{\omega }_{4},$ *and* ${\omega }_{5}$	Weight coefficients assigned to QoS parameters

### Nature‑inspired optimization algorithms

As a result of the growing popularity of nature-based optimization algorithms (metaheuristics), these algorithms have been extensively employed to deal with complex optimization problems. Their appeal can be attributed to their simplicity, flexibility, derivative-free characteristics, and nature, and they avoid local optima. These algorithms define mathematical representations inspired by natural processes. As a result, they are more straightforward and easier to synthesize when addressing real-world scenarios^34^. One of the most critical characteristics of metaheuristics is the ability to treat problems as a "black box", and so the algorithms can address a wide range of different problems without changing the algorithm structure significantly. These techniques begin with random initial solutions and avoid computing derivatives of the objective space. This renders metaheuristics suited to problems where derivative information is unknown. One of the strengths of metaheuristic algorithms is that they can avoid becoming trapped in local optima because they are stochastic. The stochasticity of algorithms enables them to escape a local solution that is not the best so far and allows for a more extensive search across the search space. The literature introduces various metaheuristic algorithms and classifies them into three classes: physics-based, swarm-based, and evolution-driven algorithms. Each class or subclass has unique properties and are able to be applied to different optimization problems.

Swarm-based techniques draw inspiration from the collective behavior observed in social organisms such as ants, bees, birds, or fish. The social species interact with one another and their environment toward a shared global goal. The central concept is to harness the emergent behavior that arises from a large group of simple agents, enabling them to solve complex problems effectively^35^. The agents' interactions lead to self-organization and problem-solving abilities at a macroscopic level, as they follow simple rules. Physics-based strategies, on the other hand, utilize the principles and laws of physics to simulate and model the behaviors of physical systems. These are often used in computer graphics, virtual reality, and engineering simulations. Physics-based methods use mathematical equations and physical properties to simulate how objects behave in the virtual environment, providing accurate and realistic representations of real-world phenomena. Nature-inspired techniques are founded on evolution-based approaches used in optimization and artificial intelligence. These methods mimic natural selection by favoring individuals with favorable traits for survival and inheritance by a mathematical function. As generations of the population evolve, the population continues to evolve and adapt within their environment. This leads to improved solutions or behaviors in relation to the task.

### Fuzzy logic

The fuzzy logic system is an interesting paradigm of artificial intelligence (AI) that was deliberately created to process unclear or uncertain information. It differs from classical logic in that classical logic operates using a binary method with either/or (true or false). Fuzzy logic can operate in degrees of truth compared to just true or false. Fuzzy logic represents variables as linguistic rather than numerical variables; linguistic variables are represented as fuzzy sets and are characterized by membership functions. The membership functions give each point of input an appropriate degree of membership between 0 and 1 and are typically expressed as a graph or curve. A fuzzy logic system is made up of fuzzy sets, fuzzy rules, a fuzzy inference engine, as well as fuzzy aggregation and fuzzy defuzzification as a methodological prerequisite for a fuzzy logic system^36^.

Fuzzy sets are sets of values with associated membership functions that describe how much a given input value belongs to each set. The membership functions may be sigmoidal, Gaussian, trapezoidal, or triangular curves. Fuzzy rules describe the relationship between fuzzy inputs and fuzzy outputs. Fuzzy logic rules typically involve "IF–THEN" sentences, where the "IF" portion sets up the conditions, and the "THEN" segment defines the actions or conclusions. These fuzzy rules are employed in the fuzzy system to manipulate incoming data to derive the satisfaction level for each rule. These rules are then defuzzified into fuzzy outputs. The fuzzy outputs are aggregated to a unique output when multiple rules are imposed concurrently using techniques like max or weighted average. After aggregation, the fuzzy outputs are defuzzified to crisp outputs. There are methods like centroid, mean of max, and weighted average that can be used for defuzzification.

### Whale optimization algorithm

**Figure 1:**
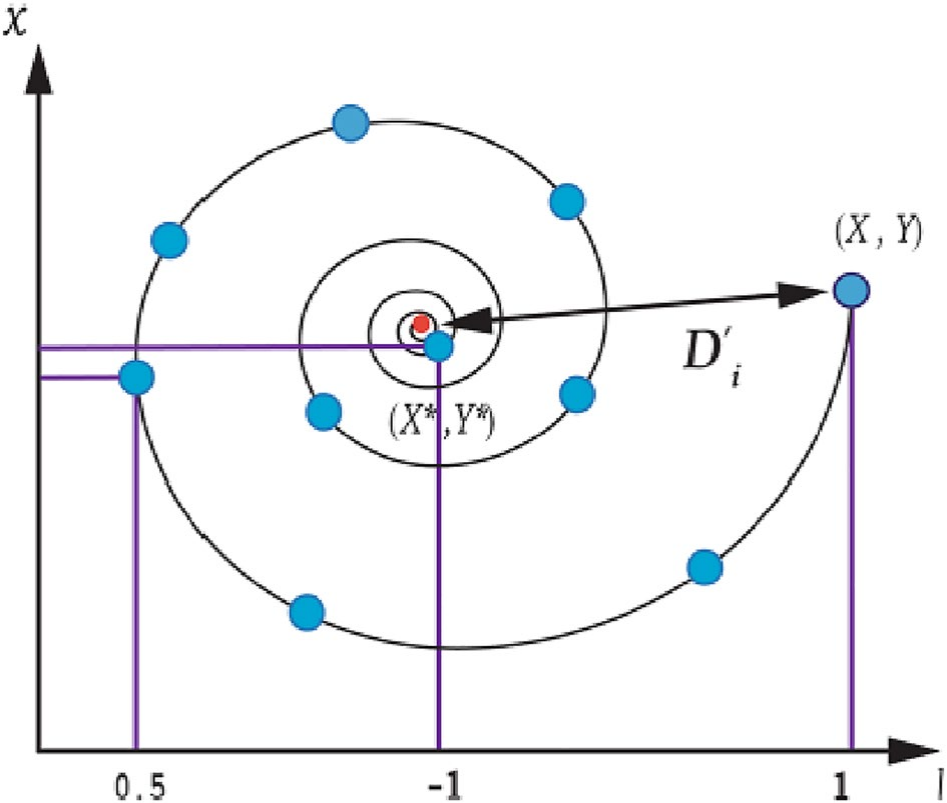
Position update in a spiral.

The Whale Optimization Algorithm (WOA) is based on the hunting strategy of humpback whales as they look for food in several dimensions. The positions of the whales are regarded as decision variables, and the food position in relation to the whale is considered as the cost function. It comprises of three main action mechanics: searching for prey, surrounding prey, and attacking the prey. Thus, hunting whales' actions will ultimately lead to its position and the present time so as to find its prey^37^. Figure 1 depicts the major presentation of the WOA. Each operational process is shown and analytically stated to aid the optimization process. The search for prey entails updating each whale's location depending on its current position and velocity. The encircling approach replicates whales working together to encircle prey and maximize the search process. By altering the placements and velocities of the whales, the diminishing surrounding prey process refines the search space even further.

Equations 1 and 2 represent whale encircling behavior during optimization mathematically. These equations describe how whales adjust their positions to mimic the encircling behavior observed around the best search agent at the time.

1 $$\overrightarrow{D}=\left|\overrightarrow{C}.\overrightarrow{{X}^{*}}\left(t\right)-\overrightarrow{X}(t)\right|$$

2 $$\overrightarrow{X}\left(t+1\right)=\overrightarrow{{X}^{*}}\left(t\right)-\overrightarrow{A}.\overrightarrow{D}$$

where *X* refers to the position vector, *C* and *A* represent coefficients, *t* represents the current iteration, and X* indicates the position vector of the best solution. X* is adjusted to find a better solution for each iteration. The coefficient vectors A and C are calculated using Eqs. 3 and 4.

3 $$\overrightarrow{A}=2\overrightarrow{a}.\overrightarrow{r}-\overrightarrow{a}$$

4 $$\overrightarrow{C}=2.\overrightarrow{r}$$

During iterations, the coefficient vector *a* gradually diminishes from 2 to 0, and the unpredictable vector *r* changes from 0 to 1. The bubble net attacking approach is used to represent the attacking behavior. Two methodologies are used to model the bubble-net behavior of humpback whales. These techniques are illustrated in Fig. 1 as the decreasing encircling mechanism and the spiral updating position mechanism. Humpback whales can use either of these strategies to capture their prey, each occurring at random with a 50% chance. A variable *p* is introduced to add randomness, with a value ranging from 0 to 1. During shrinking encircling, the value of *a* in Eq. 3 decreases gradually. After a specified number of iterations, *A* decreases gradually from two to zero within the range of [-a, a]. Humpback whales display a helix-like maneuvering pattern with their spiral positioning mechanism. The optimization algorithm exploits potential solutions during the prey attack phase. The goal of exploiting is to improve the quality of solutions found near a solution denoted *S* by searching within a limited yet promising area of the search space. As a result of this operation, the search for *S* is intensified and refined. The value of *A* ranges between -1 and 1. The best solution for all search agents is obtained if *|A|* is less than 1. Equation 5 describes the updating model.

5 $$\overrightarrow{X}\left(t+1\right)=\left\{\begin{array}{c}\overrightarrow{{X}^{*}}\left(t\right)-\overrightarrow{A}.\overrightarrow{D}\,\,if\,p<0.5\\ \overrightarrow{{D}{\prime}}.{e}^{bl}.\text{cos}\left(2\pi l\right)+\overrightarrow{{X}^{*}}\left(t\right)\,\,if\,p\ge 0.5\end{array}\right.$$

As the exploration phase begins, the search space is explored to discover potentially better solutions, resembling a global search. The search agents are mobilized during this phase based on the variation of vector *A*. The search agent will diverge significantly from the search space if *A* is set to an absolute value greater than 1. This contrasts sharply with the exploitation phase. In the exploration stage, the search agents adjust their positions based on a randomly selected search agent. In this way, different areas can be explored by adding a stochastic element to the search process. Equations 6 and 7 can be used to represent the searching mechanism mathematically.

6 $$\overrightarrow{D}=\left|\overrightarrow{C}.\overrightarrow{{X}_{rand}}-\overrightarrow{X}\right|$$

7 $$\overrightarrow{X}\left(t+1\right)=\overrightarrow{{X}_{rand}}-\overrightarrow{A}.\overrightarrow{D}$$

## System model

In this section, we will delve into various system model components, each of which plays a crucial role in the overall framework. These components include network, energy, and attack models.

### Problem statement

In contemporary WSN-based IoT scenarios, efficient management of energy resources, network communication, and data transmission is a major challenge. The dynamic and resource-constrained nature of IoT nodes, coupled with the necessity for reliable and timely data exchange, highlights the critical importance of developing novel approaches to optimize network performance while extending network lifespan. In the age of new security threats, the development of secure and reliable communication between IoT devices is particularly challenging. There is a critical need for the development of robust and adaptive solutions to address these diverse concerns, and improve the efficiency, reliability, and security in IoT networks. We propose a novel framework, FSRW, that integrates fuzzy logic-based trust assessment mechanisms into WOA. FSRW aims at reducing energy consumption, improving data transmission efficiency, and enhancing network resilience against malicious network attacks. It leverages trust-based node selection and optimization-driven clustering in IoT environments.

### Network model

**Figure 2:**
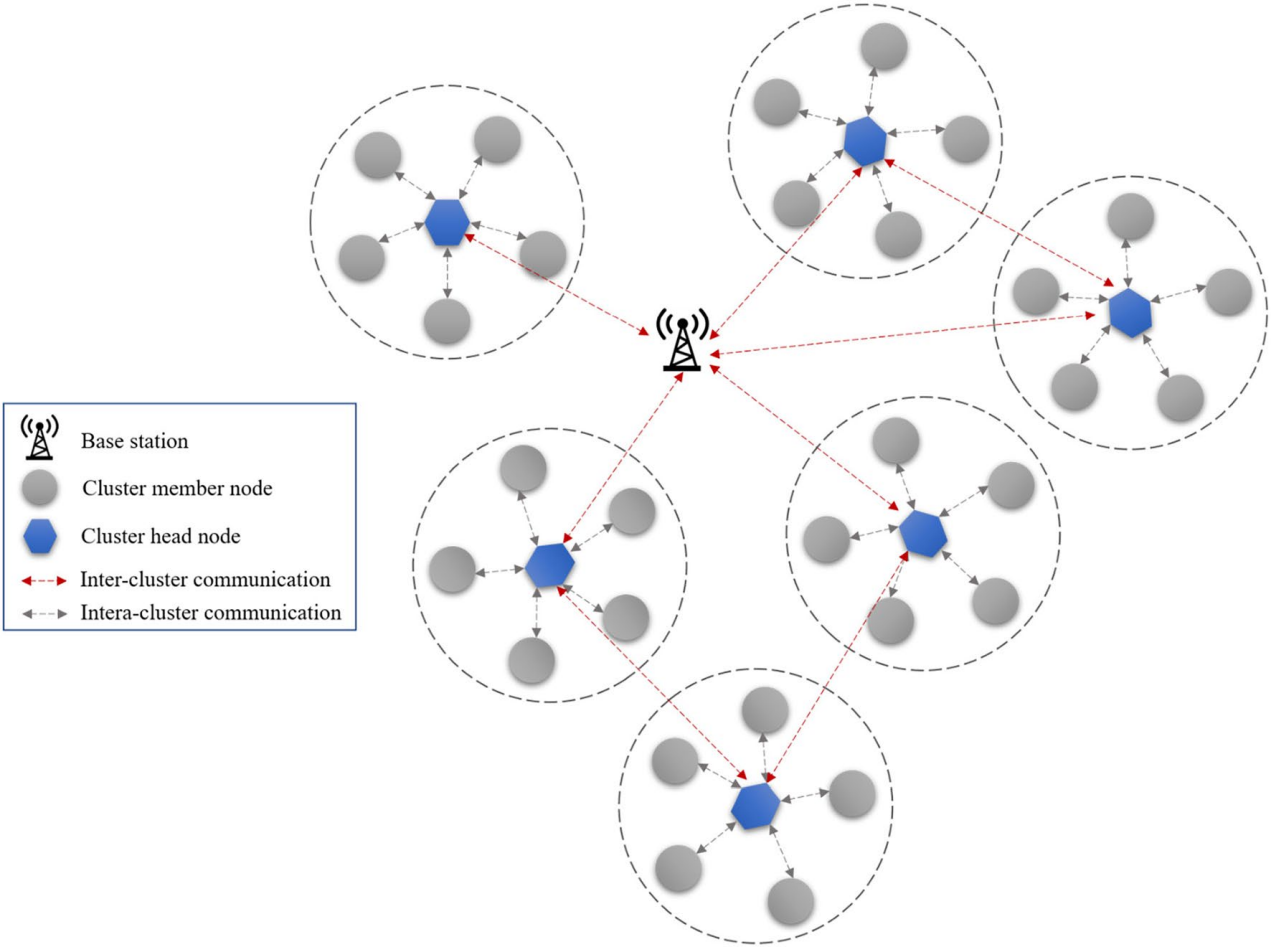
Network model.

In the FSRW, the system comprises diverse IoT nodes *(m*_*1*_*, m*_*2*_*, …, m*_*i*_*,…, m*_*U*_*)*, where *U* denotes the network's node count. Additionally, a stationary BS plays a critical role in analyzing and making decisions based on the data obtained from CHs. The BS is a central node with known positional information within the network. Each node in the network is assigned a unique identifier for identification purposes. Network nodes are distributed randomly. FSRW is designed to accommodate heterogeneous nodes, which means that different nodes possess varying energy sources, computing capabilities, and storage capacities. FSRW organizes a set of nodes into clusters, with the structure being composed of one CH and several Cluster Members (CMs) in each cluster. The CMs in each cluster have many responsibilities, such as environmental sensing and communication with the CH. The communication between nodes within a cluster is single-hop, with the CMs directly communicating with their CH. Further, the CHs are responsible for collecting, aggregating, and transmitting the data that is sensed by the CMs. The transmission of this data from CHs to the BS is multi-hop communication in which the CH eventually transmits the aggregated data to the BS after multiple hops. The communication scenarios between nodes, CHs, and the BS in the FSRW system are outlined in Fig. 2.

### Energy model

Data transfer operations involving data transmission and reception are recognized as the primary source of network energy consumption. In FSRW, energy consumption is carefully managed by considering multi-path and free space propagation modes to determine the energy usage at the sender and recipient nodes. The energy model employed in FSRW is based on a model formulated by Mohseni et al.^36^. This model calculates the overall energy expended by network nodes during data transmission and reception. The energy consumption of data transmission and reception is calculated using Eq. 8.

8 $${E}_{Total}=\sum_{i=1}^{N}({E}_{tx}^{i}+{E}_{rx}^{i})$$

The data size being exchanged is denoted by *l*. To calculate the energy used by the transmitter node during this data exchange, Eq. 9 is utilized.

9 $${E}_{tx}\left(l,d\right)=\left\{\begin{array}{c}l\times {E}_{elec}+l\times {\varepsilon }_{fs}\times {d}^{2}, d<{d}_{0}\\ l\times {E}_{elec}+l\times {\varepsilon }_{mp}\times {d}^{4}, d\ge {d}_{0}\end{array}\right.$$

In Eq. 9, several parameters are used to determine the energy consumption at the transmitter node or receiver node during data exchange. *E*_*elec*_ represents the energy required by nodes to power their electrical equipment, which includes components like transceivers and processing units. *ε*_*fs*_ stands for the free space amplification factor that takes into account the energy loss in the transmission due to signal attenuation in an unobstructed environment. *ε*_*mp*_ refers to the multi-path space amplification factor that accounts for the energy loss due to signal reflections and multi-path interference in environments with obstacles. Based on the distance between the transmitter and receiver nodes, represented by *d*, Eq. 9 calculates the consumed energy using free-space or multi-path models. The boundary condition for selecting the appropriate model is determined by the value of *d*_*0*_, obtained from Eq. 10. If the distance d is shorter than d0, the free-space model calculates the consumed energy. On the other hand, if the distance *d* is greater than or equal to *d*_*0*_, the multi-path model is employed for energy consumption calculations. Equation 10 establishes the threshold value *d*_*0*_, serving as a boundary that determines the transition between the two models. This boundary condition plays a crucial role in optimizing energy consumption during data transfer between the transmitter and receiver nodes in the network.

10 $${d}_{0}=\sqrt{\frac{{\varepsilon }_{fs}}{{\varepsilon }_{mp}}}$$

Equation 11 determines the energy consumed by the receiver node during data exchange with the transmitter node.

11 $${E}_{rx}\left(l\right)=l\times {E}_{elec}$$

### Attack model

Security is a critical concern in IoT networks that employ wireless channels for communication between nodes. The wireless nature of the network makes it vulnerable to various security threats and attacks. Malicious nodes may infiltrate the network through various means and initiate several attacks, leading to severe consequences such as disrupting normal network performance, compromising data integrity, and draining the energy of IoT nodes. Some common types of attacks considered by FSRW that can impact the secure data transfer operation in IoT networks include:

- Blackhole attack: A malicious node falsely claims to have the shortest or best path to the destination, enticing other nodes to send data packets through it. However, the malicious node drops or consumes the packets, leading to data loss.
- Sinkhole attack: An attacker sets up a malicious node that advertises itself as the sink or central data collection point. Legitimate nodes then route their data to this malicious node, causing data to be redirected and possibly manipulated.
- Wormhole attack: Attackers create a direct tunnel between two distant points in the network, enabling them to quickly transmit data packets across the tunnel, bypassing normal routing mechanisms.
- Selective forwarding: A malicious node selectively forwards only specific packets while dropping others, leading to selective data loss and affecting communication efficiency.
- Flooding attack: An attacker floods the network with excessive and unnecessary data packets, consuming network resources and potentially causing congestion and denial of service.

## Proposed approach

This section elucidates a fuzzy and secure hierarchical routing approach, referred to as FSRW, specially designed for IoT networks based on WSN technology. FSRW encompasses two primary frameworks: the fuzzy trust framework and the WOA-based clustering framework.

### Fuzzy trust framework

In FSRW, the primary objective is to determine nodes' reputations based on their interactions while transmitting and receiving data packets. This is achieved by employing a Mamdani fuzzy system to determine network nodes' trust values. The system takes two inputs, direct and indirect trust, and produces a total trust score as an output. The rule base is utilized to make trust assessments. Since nodes' trust values fluctuate due to factors such as energy depletion and node failures, IoT objects should periodically update their neighboring nodes' trust levels. The fuzzy trust framework provides a mechanism for secure routing in wireless networks, enabling nodes to evaluate the reliability of their neighbors based on their past interactions. This information is essential for making informed decisions during data packet forwarding and routing to ensure efficient and secure communication within the network. Regular updates of trust values are necessary to adapt to changing network conditions and maintain an accurate representation of node trustworthiness.

${T}_{ij}^{direct}$ represents the urgent trust generated by the *i*^*th*^ node for the *j*^*th*^ node. This urgent trust is obtained by connecting nodes *i* and *j* directly. The direct trust is determined based on various parameters, including the Consumed Energy Ratio (ECR), Packet Reception Frequency (PRF), Packet Transfer Frequency (PTF), and Packet Delivery Ratio (PDR).

The parameter *ECR*_*j*_ quantifies the energy consumption of node *j* within the time interval $[t, t+\Delta t]$. It is important to note that a higher ECRj value indicates a heightened likelihood of node *j* being potentially involved in an invasion, particularly due to the increased likelihood of a flooding event. The calculation of *ECR*_*j*_ is carried out using Eq. 12.

12 $$ ECR_{j} = \frac{{E_{j} (t) - E_{j} (t + \Delta t)}}{\Delta t} $$

In Eq. 12, *E*_*j*_*(t)* refers to the remaining energy of node *j* at time *t*, and *Ej(t* + *Δt)* represents its remaining energy at time *t* + *Δt*.

*PRF*_*j*_ indicates the number of packets received by node *j* during the period $[t, t+\Delta t]$. The higher *PRF*_*j*_ value indicates the reliability and safety of node *j*. Conversely, a low *PRF*_*j*_ suggests that node *j* might be a potential invader, as it could be susceptible to attacks like black holes, sinkholes, or gray holes. *PRF*_*j*_ is computed using Eq. 13.

13 $$ PRF_{j} = \frac{{M_{j}^{Received} }}{\Delta t} $$

In Eq. 13, ${M}_{j}^{Received}$ represents the count of packets received by the *j*^*th*^ node within the time interval $[t, t+\Delta t]$.

In Eq. 14, *PTF*_*j*_ denotes the packet count sent by the *j*^*th*^ node. A higher PTF indicates a potential for invader behavior, possibly involving flooding or wormhole attacks. This leads to a decrease in the trust value of node *j*.

14 $$ PTF_{j} = \frac{{M_{j}^{Transferred} }}{\Delta t} $$

where ${M}_{j}^{Transferred}$ refers to the total packet count sent during the interval $[t, t+\Delta t]$. Equation 15 quantifies the proportion of packets received by node *j* concerning the total number of data packets sent to that node. A higher PDR indicates the success and reliability of node *j*, reflecting its trustworthiness. In contrast, if node *j* exhibits an inadequate PDR, it implies a significant amount of missing data, which raises suspicions of potential malicious activity. This situation increases the vulnerability to attacks like black holes, sinkholes, and grey holes. Consequently, the trust value associated with node *j* decreases as a result.

15 $$ PDR_{j} = \frac{{M_{j}^{received} }}{{M_{j}^{total} }} $$

With the provided parameters, the calculation of ${T}_{ij}^{direct}$ is determined using Eq. 16.

16 $$ T_{ij}^{direct} = \frac{{\beta_{1} PRF_{j} + \beta_{2} PDR_{j} }}{{\beta_{3} ECR_{j} + \beta_{4} PTF_{j} }} $$

The weight coefficients ${\beta }_{1},{\beta }_{2},{\beta }_{3}, and {\beta }_{4}$ are adjusted within the range of *[0, 1]*, and their sum equals 1. In the FSRW approach, the calculation of ${T}_{ij}^{direct}$ is updated using window mean exponential weighted moving average. This method employs a window size to incorporate previous trust scores into the computing ${T}_{ij}^{direct}$. Therefore, the trust decision of node *i* regarding ${T}_{ij}^{direct}$ is not solely based on the current value but rather considers a set of trust values for a more informed determination. Consequently, Eq. 17 is utilized to update and refresh the value of ${T}_{ij}^{direct}$.

17 $$ T_{ij}^{direct} (l) = (1 - \beta )\frac{{\sum\limits_{k = l - w}^{l - 1} {T_{ij}^{direct} (k)} }}{w} + \beta T_{ij}^{direct} (t) $$

**Figure 3:**
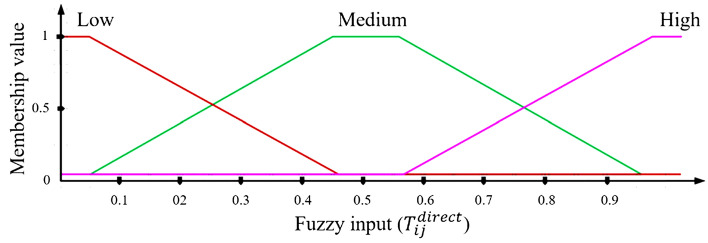
Membership function for direct trust.

Equation 10 involves the coefficient *β*, adjusted within the range of *[0, 1]*. The membership function (MF) associated with ${T}_{ij}^{direct}$ is illustrated in Fig. 3. ${T}_{ij}^{direct}$ is categorized into three levels: high, medium, and low.

The indirect trust of the *i*^*th*^ node to the *j*^*th*^ node, denoted as ${T}_{ij}^{indirect}$, represents the measure of trust derived from recommended nodes. These recommended nodes are shared and trustworthy neighbors between nodes ni and nj. These recommended nodes are selected from the pool of reliable nodes with trust values exceeding a specified threshold, denoted as *T*_*threshold*_. Let us consider a collection labeled *R* that comprises *e* recommended nodes connecting nodes *i* and *j*. In mathematical terms, we can represent this set as $R=\left\{{n}_{Recommender}^{1},{n}_{Recommender}^{2},\dots ,{n}_{Recommender}^{e}\right\}.$ With this framework, the calculation of ${T}_{ij}^{indirect}$ is accomplished using Eq. 18.

18 $$ T_{ij}^{direct} = \frac{1}{e}\sum\limits_{x \epsilon R}^{e} {\left( {T_{ix}^{direct} \cdot T_{xj}^{direct} } \right)} $$

**Figure 4:**
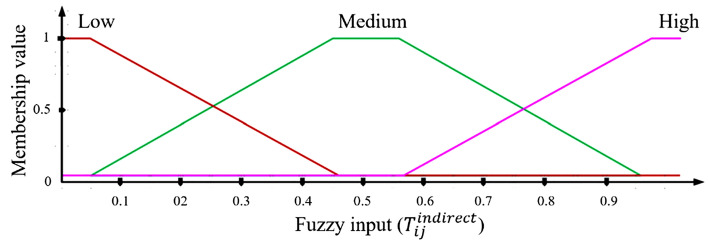
Membership function for indirect trust.

In Eq. 18, ${T}_{ix}^{direct}$ represents a direct trust between nodes *i* and *x*, and ${T}_{xj}^{direct}$ indicates a direct trust between nodes *x* and *j*. Here, *x* signifies the recommended node. The MF corresponding to ${T}_{ij}^{indirect}$ is depicted in Fig. 4, illustrating three distinct levels.

**Figure 5:**
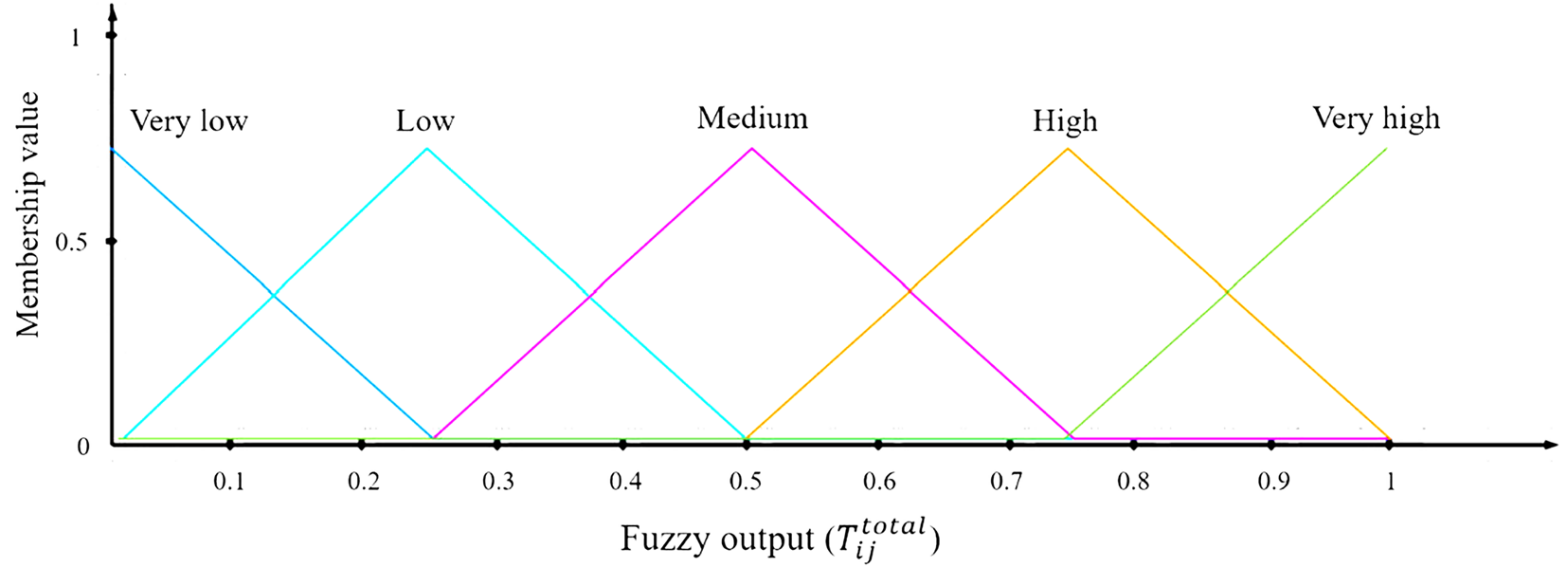
Membership function for output.

**Figure 6:**
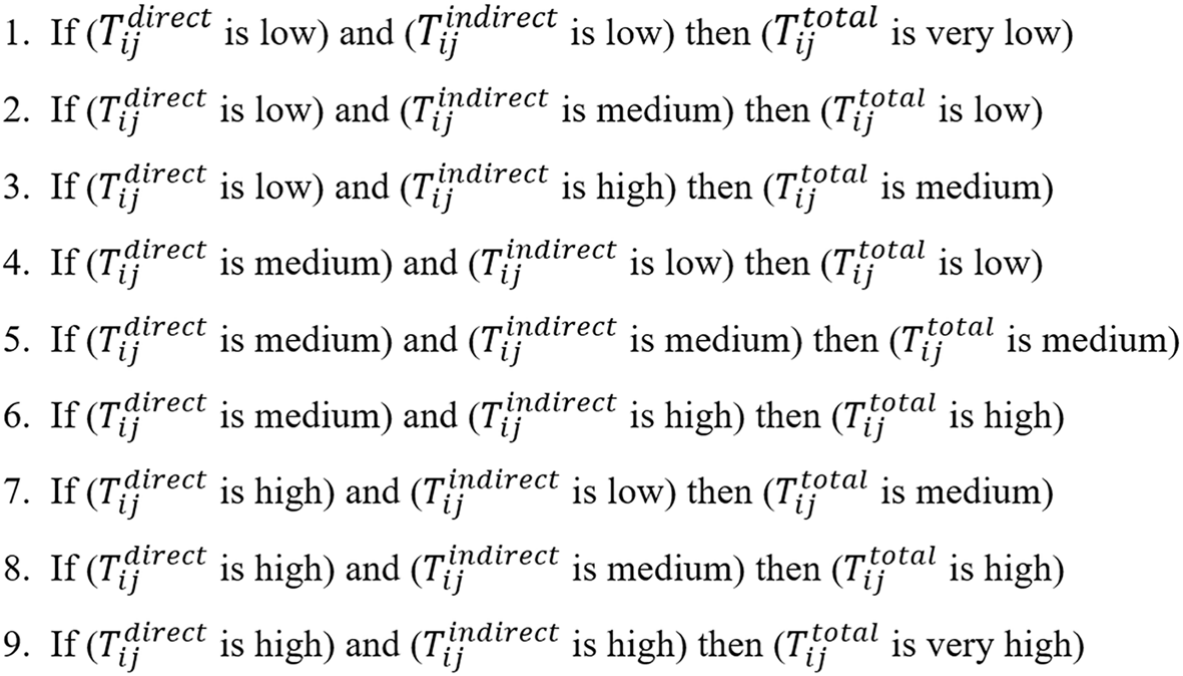
Fuzzy logic rules.

The overall trust value is the outcome of the fuzzy trust model (${T}_{ij}^{total}$). It encompasses five levels: very high, high, medium, low, and very low. The MF for ${T}_{ij}^{total}$ is visualized in Fig. 5, providing a graphical depiction of these five distinct modes. The trust framework introduced in this study summarizes a set of rules as outlined in Fig. 6.

### Whale optimization algorithm for clustering

In FSRW, the WOA-based clustering framework is outlined, which is intended to be executed by the BS. In the FSRW approach, IoT nodes, such as *i,* transmit a guiding message referred to as a "beacon" to the BS. This beacon contains essential information, including the communication range, centrality degree, hops to the BS, residual energy, position, and the trust level of nodes. Subsequently, the BS evaluates the trustworthiness of each node to distinguish between trusted and untrusted nodes. Notably, nodes with high trust values (greater than *T*_*threshold*_) are considered trusted nodes. In the FSRW framework, only trusted nodes are eligible to function as CHs. Upon identifying trusted nodes, the BS initiates the WOA-based CH selection mechanism. Whales represent IoT nodes, and the value associated with a whale signifies the probability of the node becoming a CH among neighboring nodes. Initially, whales are assigned random numbers. Nodes representing whales are candidate CHs and provide a potential solution to the CH selection issue. The key attraction of the whales is determined by ${\beta }_{0}$, which is computed using the *RAND* function. The BS bears a significant role in this CH selection framework, as it leverages the WOA to identify the optimal CHs within the network. Subsequently, the BS evaluates the fitness value of candidates (whales) in accordance with an objective criterion.

The average distance to nearby nodes (*D*_*avg*_) is a metric that indicates the centrality of node *i* within its cluster. When node *i* is situated near the center of the cluster, the value of *D*_*avg*_ is relatively low. In practical terms, by choosing a node close to the center of a cluster as a CH, the network becomes more energy efficient. This is due to the fact that selecting such a CH results in a reduced distance between CHs and their CMs. Consequently, CMs require less energy to send data packets to the CH. The following equation (Eq. 19) is utilized to quantify this parameter.

19 $$ D_{avg} = \frac{1}{{T_{i} }}\sum\limits_{u \in Nei}^{{T_{i} }} {d(n_{i} ,u)} $$

where *T*_*i*_ represents the total number of CMs associated with node *i*, *u* refers to each CM within the cluster, and *d(n*_*i*_*,u)* denotes the distance between node *i* and CM *u*.

The communication radius (*R*_*com*_) is a parameter that reflects the communication range of IoT nodes, and it can vary based on the heterogeneous nature of the nodes. In the context of the FSRW algorithm, the CH selection process considers this aspect. Specifically, nodes with a larger *R*_*com*_ are more likely to be selected as a CH since they can cover a wider area. To standardize and normalize the *R*_*com*_ parameter, Eq. 20 is employed.

20 $$ R_{com}^{norm} = \frac{{R_{com} - R_{\min } }}{{R_{\max } - R_{\min } }} $$

The number of hops to the BS (*H*_*c*_) is a crucial factor in the CH selection process as it significantly influences the decision-making for CHs. Nodes with fewer hops to the BS are preferred as CHs because they enable faster data transmission to the BS, resulting in reduced latency and improved efficiency in the network. To standardize and normalize the *H*_*c*_ parameter, Eq. 21 is utilized:

21 $$ H_{c}^{norm} = \frac{{H_{c} - H_{\min } }}{{H_{\max } - H_{\min } }} $$

The remaining energy (*E*_*r*_) parameter serves a crucial role in the objective function, aiming to achieve a balanced distribution of energy consumption among IoT nodes and consequently prolong the overall network lifetime. The intention is to ensure that high-energy nodes are responsible for becoming CHs since CHs bear greater tasks and consume more energy. Allowing low-energy nodes to become CHs could deplete their energy quickly, leading to the need for frequent CH replacements, which incurs additional costs, time, and communication overhead. *E*_*r*_ is extracted from the guide message from node *i* by the BS. The energy aspect holds significant weight in the decision-making process for selecting CHs, given the energy constraints of IoT nodes. Since nodes within the network possess varying energy levels, if a particular node, such as *i*, has higher energy reserves than others, it is granted a higher probability of being chosen as a CH. The normalization of *E*_*r*_ is achieved through Eq. 22.

22 $$ E_{r}^{norm} = \frac{{E_{r} - E_{\min } }}{{E_{\max } - E_{\min } }} $$

Consequently, the objective function is computed based on Eq. 23:

23 $$F=\frac{{\omega }_{1}{R}_{com}^{norm}+{\omega }_{2}{E}_{r}^{norm}+{\omega }_{3}{T}_{norm}^{total}}{{\omega }_{4}{D}_{avg}^{norm}+{\omega }_{5}{H}_{c}^{norm}}$$

${\omega }_{1}, {\omega }_{2},{\omega }_{3},{\omega }_{4},$ and ${\omega }_{5}$ ​are the weight coefficients for the respective parameters. This objective function is the cornerstone of the decision-making process in the FSRW algorithm for selecting CHs within the IoT network. By incorporating multiple parameters and assigning appropriate weights to them, the algorithm aims to optimize the selection of CHs based on criteria such as trust, centrality, communication range, distance to the BS, and energy availability. The weights ${\omega }_{1}, {\omega }_{2},{\omega }_{3},{\omega }_{4},$ and ${\omega }_{5}$ allow fine-tuning the significance of each parameter in the CH selection process, ensuring a balanced consideration of various factors for improved network performance and longevity.

Upon calculating the probability of whales (representing IoT nodes), the positions of these whales are updated. Within the WOA-based CH selection model, the algorithm iterates 300 rounds as a stopping point. Once the WOA-based clustering process concludes, the BS selects the most efficient whale (IoT node) with the best fitness value as the CH. Subsequently, the BS communicates the roles to the IoT nodes. The CHs generate notification messages to inform their neighboring nodes about their designated roles in the network. These notifications contain the coordinates of the CHs. Upon receiving these notifications, the neighboring nodes undertake specific actions based on different scenarios. In the first scenario, if an ordinary node receives one or more notification messages from various CHs, it sends a membership request to the nearest CH, expressing its intent to join that cluster. In the second scenario, if an ordinary node possesses a trust level exceeding the predefined threshold (*T*_*threshold*_) and does not receive any notification messages from CHs, it assumes the role of a CH itself. In this case, the node broadcasts a notification message to announce its new role and identity. As a result of these processes, the network now consists of two distinct types of nodes: CHs and CMs. CM nodes are exclusively associated with their respective CHs and forward their data exclusively to them. On the other hand, CHs maintain direct communication with their CMs and neighboring CHs, enabling efficient data transfer and management within the clustered network structure.

Cluster maintenance is crucial to ensure stable network connections and roles for each node through periodic guide message exchanges. This process involves several key steps to manage and adjust the roles of IoT nodes within the clusters. All IoT nodes actively engage in this ongoing procedure, which encompasses the following stages:

- Connect to the cluster: Whenever a new IoT node joins the network, it initiates the process by broadcasting a membership request. A CH that receives this request first responds promptly and acknowledges the new node's membership in its cluster.
- Leave the cluster: Periodic exchange of guide messages enables each CM to assess its connection status with its respective CH. If this link is inactive or invalid, the CM is disassociated from the cluster. Subsequently, the CM broadcasts a membership request across the network to connect with the nearest available CH.
- Cluster membership verification: CHs regularly evaluate their communication links with associated CMs employing periodic guide message exchanges. If any communication links are deemed ineffective, the CH takes the necessary steps to revoke the membership of the concerned CM from its cluster.
- Re-clustering: Over time, IoT nodes' status may change according to factors such as trust values or energy usage. To address these changes, the BS continually monitors the status of nodes through updates sent by the IoT nodes. If a CH's trust value or energy level falls below a certain threshold, it sends a cluster update message to its CMs. This triggers a re-clustering process, prompting the CHs to initiate a new round of the WOA-based selection framework. During this time, IoT nodes await the BS to execute the WOA-based clustering operation, enabling the selection of a new CH.

Through these steps, the cluster maintenance process ensures the network's stability, reliability, and adaptability by efficiently managing the connections and roles of IoT nodes within the clusters.

**Algorithm 1:**
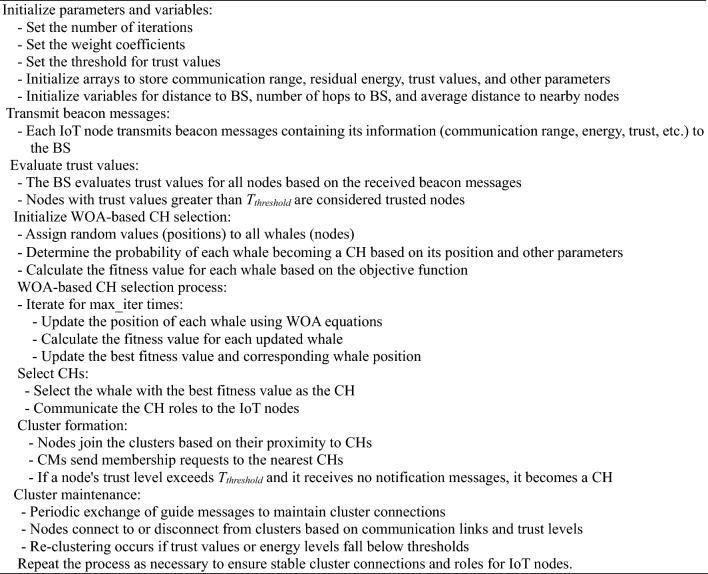
Pseudo-code for WOA-based clustering

## Findings and discussion

A thorough assessment of the FSRW entails detailed simulation and evaluation in various scenarios. To achieve this objective, the simulation processes are performed using the Network Simulator 2 (NS2) software. The simulation area size is 100 × 100 m^2^. In this simulation scenario, 100 nodes are randomized and placed in the area. These IoT nodes are stationary in the simulation area. The energy profiles of the nodes are heterogeneous. The transmission radius of these nodes is established at 20 m. In adherence to the FSRW model, a supposition is made that 10% of the nodes harbor hostile intentions. The subset of hostile nodes is chosen by chance from the complete range of network nodes, including nodes with distinct energy levels. In each iteration of the simulation, 100 transmission operations are performed, with each packet comprising 500 bytes. This part of the research entails the evaluation of five distinct performance metrics, which encompass the Packet Delivery Rate (PDR), energy equilibrium, energy level assessment, network lifespan, and trustworthiness status. The simulation parameters are summarized in Table 3.

**Table 3:** Simulation parameters for FSRW evaluation.

Parameter	Description
Simulator	Network simulator 2 (NS2)
Simulation area	100 × 100 m^2^
Number of nodes	100
Node mobility	Stationary
Energy profile	3 nodes: 6 J, 12 nodes: 5 J, 35 nodes: 4 J, and 50 nodes: 2 J
Transmission radius	20 m
Percentage of hostile nodes	10% of the total nodes
Packet size	500 bytes
Transmission operations	100 per iteration
Performance metrics evaluated	PDR, energy equilibrium, energy level assessment, network lifespan, and trustworthiness status

**Figure 7:**
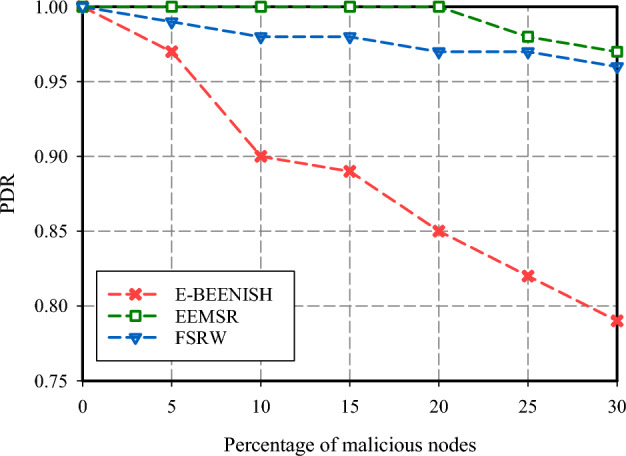
PDR comparison.

**Figure 8:**
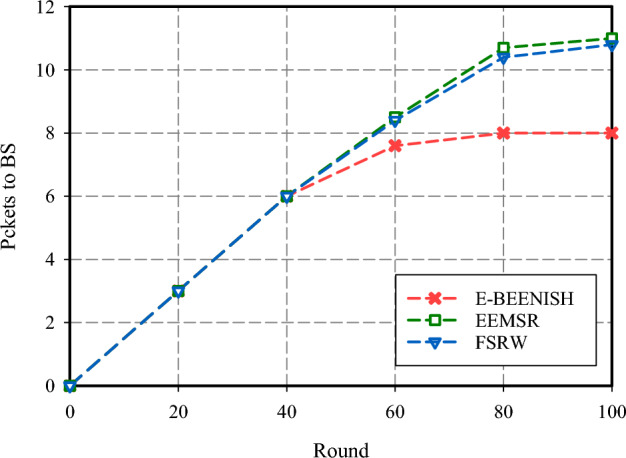
Packets routed to the BS comparison.

The initial evaluation parameter for assessing the performance of the FSRW method pertains to the investigation of PDR within the network. The findings of this assessment are illustrated in Figs. 7 and 8. The outputs of PDR in response to different fractions of hostile hosts within the network are shown in Fig. 7. The relative PDR shown by FSRW is less (about 1%) than EEMSR, which is indicative of a more efficient reliance mechanism than EEMSR. In addition, FSRW demonstrated a gain in PDR (about 17.7%) compared to E-BEENISH. The main reason for this gain is the focus on network security in FSRW. On the contrary, the E-BEENISH system experiences a rapid decline in PDR as the proportion of malicious nodes within the network increases. Figure 8 gives insight into the number of data packets successfully routed to their BS, which is equivalent to their destination. As shown in Fig. 8, FSRW provides lower PDR than EEMSR.

**Figure 9:**
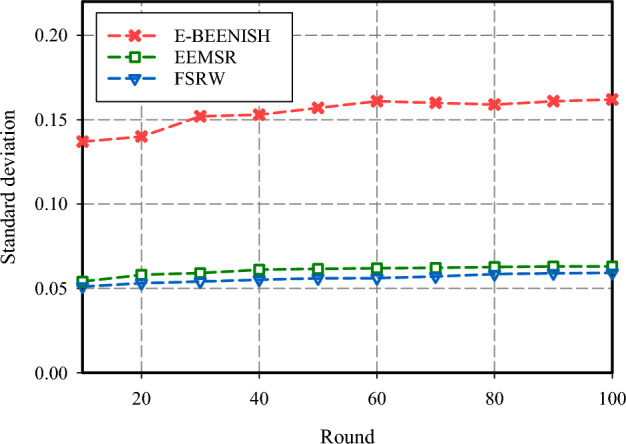
Energy equilibrium comparison.

Energy equilibrium is crucial in assessing FSRW efficiency. In order to evaluate energy balance, the Standard Deviation of Energy Consumption (*SD*_*Energ*_) metric is computed for every node. The results of the assessment are depicted in Fig. 9. A smaller number for *SD*_*Energ*_ would be indicative of more balance in energy consumption among the nodes, while a larger number towards one indicates more imbalance. As shown in Fig. 9, FSRW has the lowest value for *SD*_*Energ*_, and hence is the most balanced in terms of energy consumption among the network nodes.

**Figure 10:**
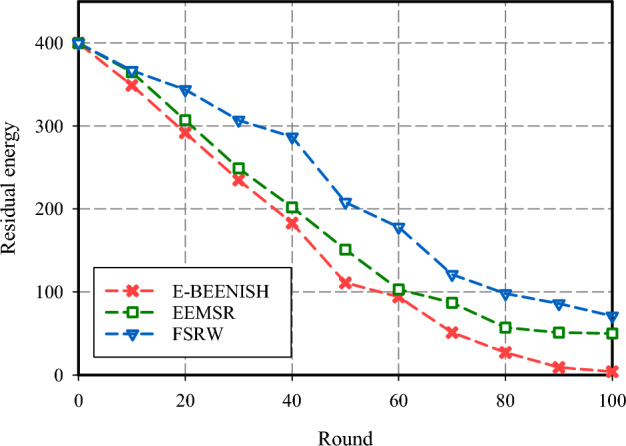
Residual energy comparison.

Evaluating energy distribution across network nodes is another major benchmark. This assessment is depicted in Fig. 10. The results suggest that FSRW has shown a great improvement in terms of stored energy across the nodes resulting in 78% and 29% better stored energy compared to E-BEENISH and EEMSR respectively. Looking at Fig. 10 shows that FSRW and EEMSR have done good work in terms of energy preservation across the network. On the other hand, E-BEENISH has done less well at preserving energy across the network. The main reason behind this issue is that E-BEENISH has not been guided regarding node energy during CH selection. For the CH to BS under the E-BEENISH, the transmission of data is done directly which results in decreased energy stored in the nodes.

**Figure 11:**
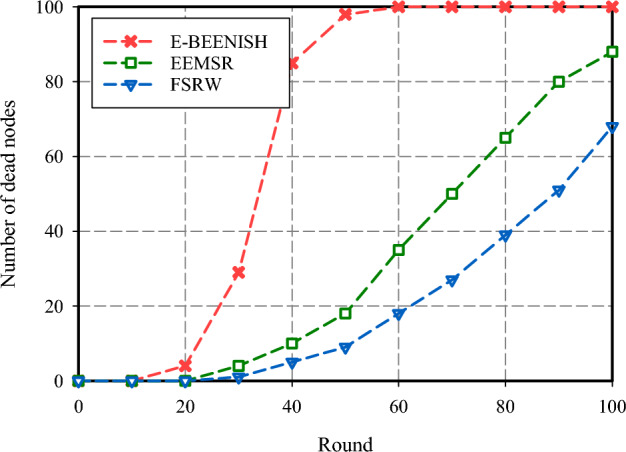
Network lifespan comparison.

**Figure 12:**
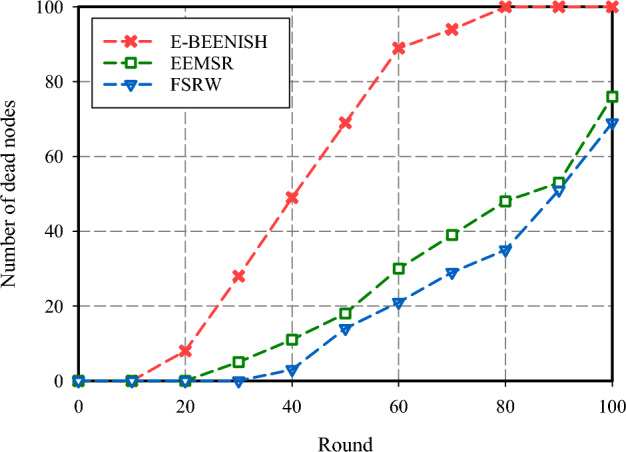
Network lifespan comparison (BS positioned at coordinates (0, 0).

**Figure 13:**
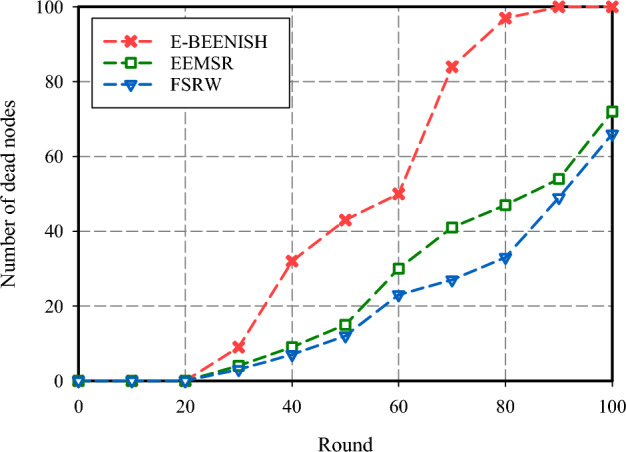
Network lifespan comparison (BS positioned at coordinates (50, 50).

The fourth criterion used to evaluate the performance of FSRW focuses on the network's longevity, measured by counting the number of idle nodes in each cycle. As illustrated in Fig. 11, FSRW has the most resilient network lifespan, with approximately 29% more surviving nodes than EEMSR and significantly outperforming E-BEENISH at nearly 47% more nodes per round. It is noteworthy that the performance of EEMSR and FSRW is closely aligned when considering network longevity based on the first node's cessation of operation. However, divergent results emerge when evaluating network longevity relative to one-half of network nodes failing or the loss of the last node. Another aspect of the assessment includes relocating the BS to different locations and measuring its effect on routing metrics. In Fig. 12, the BS is situated in the corner, at the coordinate (0, 0). It shows that FSRW has largely increased the number of inactive nodes at each round over E-BEENISH (44%) and EEMSR (11%). As shown in Fig. 13, the BS is at the center, at the coordinate (50, 50). Also, it has proven that FSRW have improved this metric over EEMSR (14.50%) and E-BEENISH (66.65%). Importantly, the experiments underscore that altering the BS position within FSRW and EEMSR has a relatively minor impact on their respective performance.

**Figure 14:**
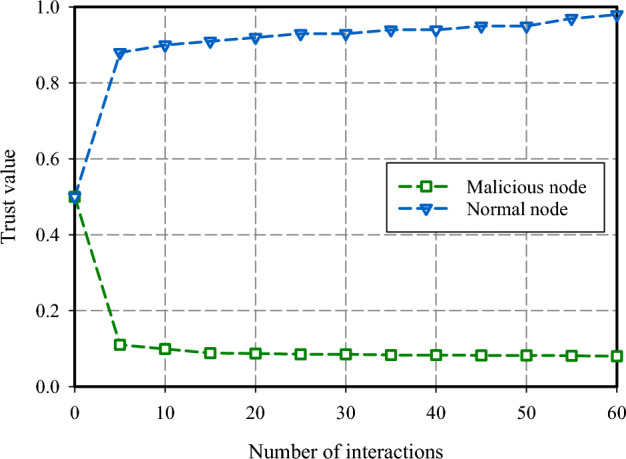
Trustworthiness status comparison.

To analyze FSRW performance, the trust status evaluation of the nodes is performed as a final parameter. The analysis visualization is shown in Fig. 14. Two scenarios of the evaluation process are outlined, where we consider setting the initial trust value of the nodes to 0.6 and having intrusive entities in 10% of the network. Based on the results in Fig. 14, distinguishing intruder nodes from honest nodes is challenging at the beginning of network deployment given the limited information exchanges and immature trust level understanding. The fuzzy trust mechanism in FSRW allows the establishment of trust between the node and the neighbors under the scenario of more communication and information being exchanged among the nodes. Once the communication frequency is increased, the trust value of a trustworthy node is increased to 1, and the trust value of the intruder node is reset to 0 by the FSRW fuzzy trust scheme. Thus, it sufficiently separates out the intruder nodes from the honest nodes, as explained in Fig. 14.

## Conclusion

This paper presented a novel approach to enhance routing efficiency and security in IoT-based healthcare systems by integrating WOA and fuzzy logic-based trust assessment. The proposed FSRW framework addressed the critical challenges of ensuring reliable and secure data transmission while minimizing energy consumption. FSRW assessed the trustworthiness of resource nodes through both WOA and a Fuzzy Trust framework, ensuring routing attack prevention and network integrity. The WOA based clustering framework added to the excellence of CH node selection, selecting the CH node based on various factors including centrality, communication range, hop count, remaining energy and trustiness. We have presented an integrated framework to ensure the security, stability, and lifecycle of the IoT-based health network. FSRW is typically superior to EEMSR and E-BEENIS routing protocols in terms of network lifetime and the packet delivery ratio. The findings endorse the efficiency of FSRW in preserving network operational lifetime, energy utility, and seamless information packets. By combining WOA and trust assessment based on fuzzy logic, the FSRW provides a new benchmark for routing protocols in healthcare systems, leading the way for robust, secure, and efficient communication networks. Based on the discussed research, possible future directions may be to investigate the use of IoT network and the machine learning algorithms to enhance the trust assessment of the IoMT networks, as well as the scalability of FSRW under the largescale network and the extension of the application field to various domains beyond the healthcare domain together with their own frameworks and methodologies for the potential applications in the future.

## Data Availability

The experimental data used to support the findings of this study are available from the corresponding author upon request.
